# Amniotic fluid stem cells: A novel treatment for necrotizing enterocolitis

**DOI:** 10.3389/fped.2022.1020986

**Published:** 2022-12-01

**Authors:** Felicia Balsamo, Yina Tian, Agostino Pierro, Bo Li

**Affiliations:** Division of General and Thoracic Surgery, Translational Medicine Program, The Hospital for Sick Children, Toronto, ON, Canada

**Keywords:** necrotizing enterocolitis (NEC), amniotic fluid stem cells (AFSCs), stem cell, translational research, preclinical study

## Abstract

Necrotizing enterocolitis (NEC) is a gastrointestinal disease frequently prevalent in premature neonates. Despite advances in research, there is a lack of accurate, early diagnoses of NEC and the current therapeutic approaches remain exhausted and disappointing. In this review, we have taken a close look at the regenerative medical literature available in the context of NEC treatment. Stem cells from amniotic fluid (AFSC) administration may have the greatest protective and restorative effects on NEC. This review summarizes the potential protection and restoration AFSCs have on NEC-induced intestinal injury while comparing various components within AFSCs like conditioned medium (CM) and extracellular vesicles (EVs). In addition to therapeutic interventions that focus on targeting intestinal epithelial damage and regeneration, a novel discovery that AFSCs act in a Wnt-dependent manner provides insight into this mechanism of protection. Finally, we have highlighted the most important aspects that remain unknown that should be considered to guide future research on the translational application of AFSC-based therapy. We hope that this will be a beneficial frame of reference for the guidance of future studies and towards the clinical application of AFSC and/or its derivatives as a treatment against NEC.

## Introduction

Necrotizing enterocolitis (NEC) is a gastrointestinal disease frequently prevalent in preterm neonates. Of those, 5%–12% of very-low birth weights are at an increased risk (1). Risk factors include intestinal immaturity, impaired microvascular circulation, abnormal intestinal microbiota, and a highly immunoreactive intestinal mucosa which may lead to necrosis (1–3). With advances in the management of preterm neonates, the overall incidence of NEC has decreased from 6.6% in 2007 to 3.9% in 2013 (4). However, there remains a difficulty in accurate, early diagnoses, and innovative treatment of NEC resulting in 27%–52% of very-low birth weight infants with NEC developing advanced NEC and requiring surgical intervention and approximately half of the infants developing neurodevelopmental damage (5). The mortality of advanced NEC (30%–50%) remains one of the highest in neonatology (4). The impact NEC has on the overall quality of life of patients and families is detrimental as diagnosis, treatment, long-term outcomes, and the cumbersome financial burden have remained stagnant throughout the past decade, despite improvements in neonatal intensive care. The cost of ongoing treatment has averaged to be $500 million to $1 billion per year in the United States (6).

In recent years, investigation into preventative actions have shown to be promising in reducing the incidence, morbidity and mortality of the disease (3). Breast milk is a significant factor in countering the occurrence of NEC (7). Research has identified components of the breast milk including milk-derived exosomes (8, 9) and human milk oligosaccharides (HMOs) (10) that regulate this beneficial effect. The use of prophylactic probiotics, prebiotics, and synbiotics administered to premature neonates is another plausible strategy to prevent the onset of NEC (11). Lastly, recent advances in remote ischemic conditioning (RIC) application may prevent the progression of NEC, avoiding irreversible damage to the neonate (12). However, the exact pathophysiology and “curative” treatment strategy remains unknown. Therefore, experimental and clinical research continue to investigate the pathogenesis of NEC and develop novel treatments to avoid disease progression and alleviate morbidity and mortality.

The pioneering of regenerative medicine has become a novel tool in understanding the pathogenesis of many diseases and development of treatment strategies (13). Their mechanism of action *via* cell surface markers and release of transcription factors grants self-renewal capabilities (14, 15). The administration of stem cells can regenerate tissue administered in experimental and clinical settings. In inflammatory bowel disease (IBD), evidence demonstrates successful autologous stem cell transplantation and diminished intestinal damage (16).

Recent research has suggested that stem cells, specifically amniotic fluid stem cells (AFSCs), can potentiate the treatment effects of experimental NEC. In this article, we will review the current evidence and discuss how promising regenerative medicine and stem cell therapy may be on the clinical translation of AFSC-based therapy against NEC. Moreover, we have highlighted aspects that remain unknown, which should be considered to guide future research of the translational application of AFSC-based therapy.

## Discovery of amniotic fluid stem cell potential in NEC

### Amniotic fluid in the context of NEC

During gestation, the fetus is encapsulated by amniotic fluid (AF), which is principally composed of water and solutes (17). These solutes include a variety of trophic factors, cytokines and growth factors that modify intestinal nutrient absorption and development, increase enterocyte proliferation, migration and differentiation, prevent apoptosis and promote mucosal restoration. Ultimately, these solutes reduce intestinal injury and aid in the protection against NEC (18). In addition, AF contains antimicrobial proteins and peptides (APPs) that support the microbial activity observed. Though often discarded as “biological waste”, due its properties described, AF has been hypothesized to protect the fetus from gastrointestinal impairment and supports stable growth and differentiation. It has been reported that Toll-like receptor 4 (TLR4) activation leads to mucosal injury *via* increased enterocyte apoptosis in NEC (19). Interestingly, in 2012, Good et al. demonstrated that AF inhibits TLR4 signalling in the neonatal intestinal epithelium (20). This study provides the foundation to amniotic fluid's future application in NEC prevention and treatment.

### Discovery of amniotic fluid stem cell potential in NEC

Evidence of the potential of stem cell therapy against intestinal diseases has been a growing area of research including NEC. Adult stem cells, such as the mesenchymal stem cells (MSCs), were of the first to demonstrate protection from experimental NEC in rats in 2011 (21).

**Figure 1:**
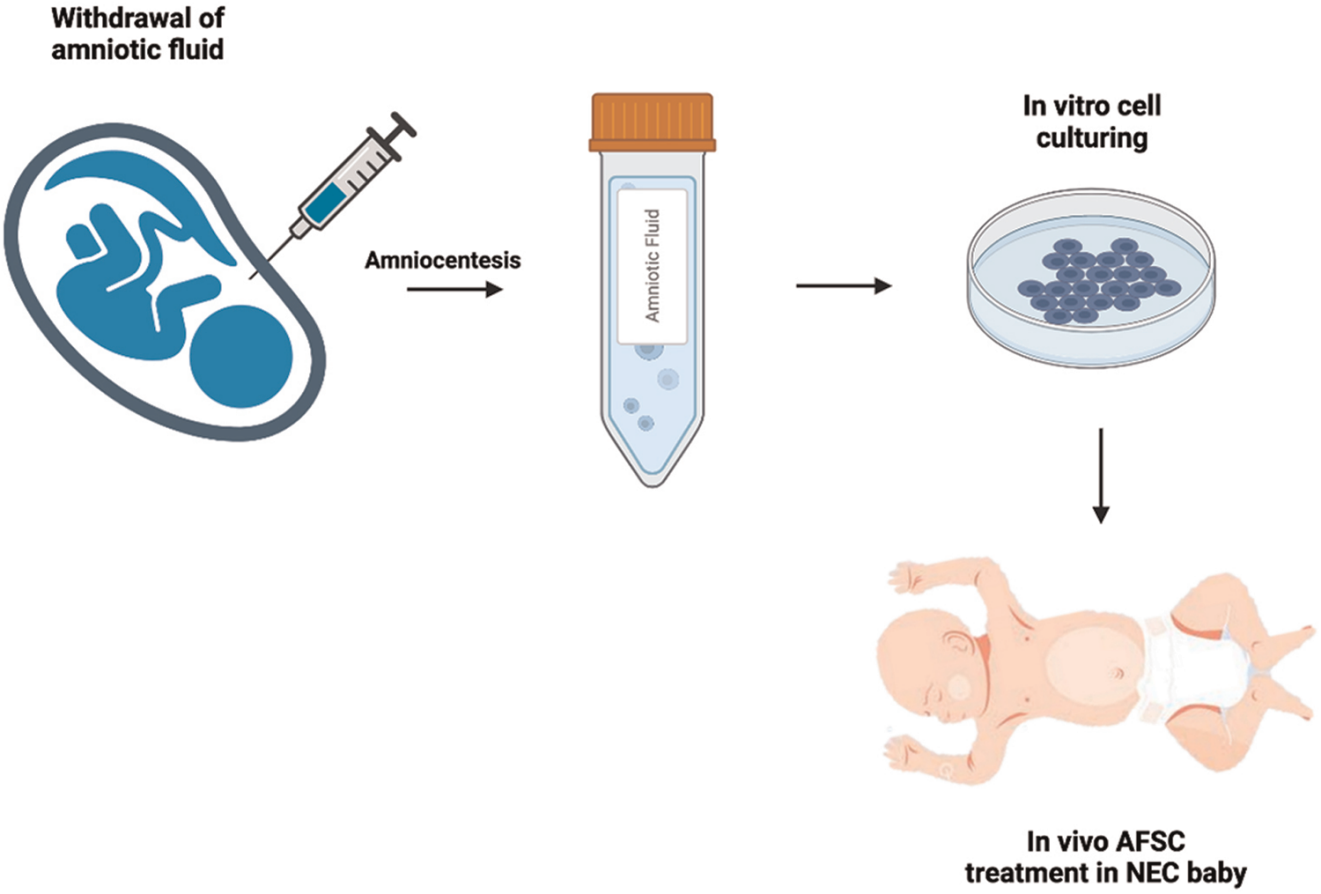
Schematic of AFSC collection and administration. Following amniocentesis, amniotic fluid stem cells are cultured from the amniotic fluid, then administered to the neonate suffering from NEC.

Moreover, De Coppi et al. discovered a stem cell derived from amniotic fluid in 2007 (22) (Figure 1). These cells demonstrated pluripotency through the confirmation of Oct-4, a marker for pluripotent human stem cells (23). The pluripotent nature of these cells include: the ability to differentiate into the three germ layers *in vitro*, to be cultured infinitely in an undifferentiated state, and to produce clonal lineages and teratomas *in vivo* (24, 25). Remarkably, AFSCs do not have the same detrimental effects as embryonic stem cells (ESCs) and adult stem cells (26). Moreover, they show greater immunosuppressive function and are capable of greater maintenance of pluripotency (27). Furthermore, AFSCs are not bound by the legal and ethical limitations that ESCs are subject to. They prove to be the more attractive stem cell for clinical use due to their greater differentiation capacity and plasticity, lower immunogenicity, and lack of tumorigenicity and ethical concerns (28).

Extraordinarily, AFSC-based therapy against NEC was first discovered by Zani et al. in 2013 (25). AFSCs injected in experimental NEC successfully integrated into the bowel wall improving survival, clinical status, and gut structure and function. Moreover, it was shown that AFSCs function *via* a cyclooxygenase 2 (COX-2) dependent mechanism. These unique therapeutic effects and promising potential launched the focus on AFSC-based therapy against NEC.

### Direct comparison of AFSC and other stem cells in NEC

Several experimental animal NEC studies have been conducted to identify the efficacy of different types of stem cells, such as amniotic fluid-derived stem cells (AFSC), bone marrow-derived MSC (BM-MSC), or enteric neural stem cells (E-NSC). McCulloh et al. compared these stem cell types and found that, generally, all demonstrate a reduction in the incidence and severity of NEC (29, 30). In addition, they found a similar rescue of barrier function in the injured intestine (29). However, the individual mechanisms of action remain unknown. AFSC are fibroblast-like, pluripotent stem cells with a great capacity for differentiation and immunomodulation (31). Their amniotic fluid origin renders them less tumorigenic and immunogenic compared to the other stem cells. BM-MSC function is dependent on modulation of stromal cells; however, these BM-MSC lack the potential to be effective in prolonging survival in the experimental NEC model (25, 32). E-NSC are stem cells that are a part of the enteric nervous system (ENS) that differentiate into neurons and glial cells (33). It has been proposed that ENS immaturity may predispose premature neonates to NEC, but with neuro-transplantation of E-NSCs, the injured ENS can be rebuilt.

Both AFSC and MSC have been investigated as potential treatment strategies in lowering NEC incidence and reducing the intestinal inflammation. However, AFSCs, not MSCs, administered prior to the onset of NEC have beneficial effects on prevention of intestinal epithelial injury (34), which is in line with a previous study comparing AFSCs and MSCs (30). In addition, AFSCs can “home” in on the intestinal epithelia while MSCs fail to do the same (25). Proteomics analysis of both AFSCs and MSCs suggested that AFSCs are primarily involved in cell development and growth, while MSCs are more immunomodulatory within the gut. These findings further support previous evidence that MSCs are effective in other inflammatory gastrointestinal diseases (35, 36). The protective effects of AFSCs in reducing intestinal damage were accompanied by increasing epithelial cell and intestinal stem cell proliferation, which results in epithelial regeneration. Remarkably, AFSCs were found to protect a healthy gastrointestinal tract with unseen damage compared to MSCs. These findings present AFSCs to be the more protective and attractive option for clinical use in NEC *via* stable intestinal proliferation, multipotency, and immunomodulatory abilities.

## Mechanism of action: amniotic fluid stem cell potential in NEC

**Figure 2:**
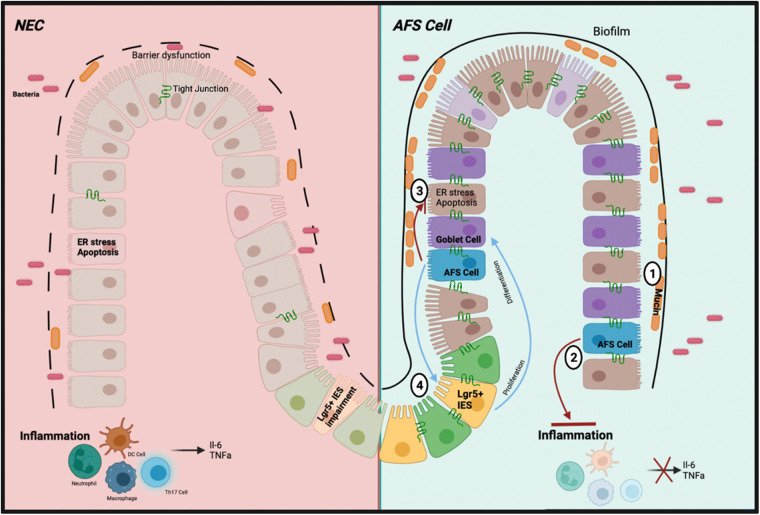
Schematic of the mechanism of action of AFSC in preclinical study. (**1**) Increased production of mucin levels by goblet cells due to increased differentiation (**2**) Inhibited inflammatory pathways and subsequent cytokines (**3**) Inhibited ER stress response and subsequent apoptosis (**4**) Restored function of ISCs to induce cell proliferation and differentiation.

Upon retrieval of the AF from the pregnant mother donor *via* amniocentesis, the AFSCs are isolated in cultured medium then administered *via* an intraperitoneal injection. The AFSCs can integrate into the injured bowel acting on the damaged intestinal area due to its preferential attraction to areas of intestinal injury. There are various modes of action by AFSCs (Figure 2); to elucidate these effects we refer to the various elements of NEC pathophysiology. The development of NEC includes intestinal barrier dysfunction, ER stress associated apoptosis, inflammation, and decreased mucosal regeneration. AFSCs can restore lost function and rescue the injured intestine.

### Barrier dysfunction

The intestinal epithelial injury seen in NEC involves a weakened integrity of the epithelial barrier, thus allowing for the translocation of potentially harmful intestinal bacteria into circulation, leading to the inflammation observed (37). The epithelial barrier contains tight junctions (TJs) which serve as a blockade for control over the translocation of microbial products into the gut wall (38). Claudin 2 is an ileum TJ protein important in the paracellular transport of cations and water. A previous study found this TJ protein to be elevated in the urine and decreased in the intestinal tissue of neonatal NEC patients, creating selective pores within the epithelial wall contributing to the weakened integrity of the epithelial barrier. Moreover, Claudin 7, another TJ protein, is an important indicator of intestinal barrier injury and decreased expression is exhibited in NEC (39). With this knowledge, a recent study identified further insight into pathogenesis by experimentally attenuating disrupted intestinal barrier function during NEC-induced intestinal injury *via* administration of AFSCs (40).

### Endoplasmic Reticulum (er) stress and its induced apoptosis

The ER stress response becomes activated upon accumulation of misfolded or unfolded proteins leading to decreased synthesis of functional proteins (41). To cope with ER stress, an adaptive response attempts to restore protein homeostasis and ER function *via* the unfolded protein response (UPR), governed by the ER stress central regulatory protein binding immunoglobulin protein (BiP). BiP controls three downstream ER transmembrane stress sensor proteins: inositol-requiring enzyme 1α (IRE1α), activating transcription factor 6 (ATF6) and pancreatic endoplasmic reticulum kinase (PERK). Furthermore, BiP activates these proteins with anti-apoptotic properties and promotes cell survival. However, prolonged ER stress activates the C/EBP homologous protein (CHOP), a part of both pro- and anti-apoptotic pathways which include encoding genes within the BCL2-family proteins. Since NEC is associated with increased intestinal epithelial cell and TJ apoptosis, inhibiting apoptotic signaling *via* CHOP activation may rescue barrier function (42).

It has been shown that ex vivo intestinal organoid model of NEC epithelial permeability and TJ disruption following NEC-induced injury were prevented by AFSCs (40). Claudin 2 expression was upregulated, while Claudin 7 was downregulated. In addition, the three ER stress sensor proteins (ATF6, IRE1α, PERK) were elevated in these AFSC-treated organoids, thus leading to the upregulation of activating transcription factor 4 (ATF4), which is important in the activation of CHOP and the pro-apoptotic pathway (40). Interestingly, the central controller, BiP, remained unaffected. Further, Li et al. analyzed whether the epithelial barrier restoration is dependent upon ER stress activity through administration of HA15, an inhibitor of the ER stress response, to the organoids (40). Any rescue observed by AFSCs was reverted to NEC injury phenotype, therefore AFSC-mediated intestinal barrier protection is dependent on the ER stress response. Remarkably, cell necrosis and apoptosis were reduced following AFSC treatment. Thus, AFSC-mediated anti-apoptotic effects are dependent on the ER stress response. In an *in vivo* NEC model, TJ function was restored, rescuing intestinal barrier function (40). Like the ex vivo model, AFSC-mediated ER stress was activated, while sequestering apoptotic effects. This differential effect of the ER stress response occurs because of different intestinal damage intensity points. When damage is at its highest, ER stress markers that would normally increase apoptosis decline. AFSCs proves to rescue intestinal permeability in experimental NEC *via* AFSC-induced ER stress without inducing apoptosis.

### Immunological regulations

Intestinal inflammation is another common marker of NEC, which is closely correlated with barrier dysfunction, ER stress, and apoptosis. Upon intestinal injury, endotoxins released from intra-luminal bacteria bind to Toll-like receptor 4 (TLR4) present on intestinal epithelial cells (3). TLR4 expression is increased on intestinal epithelial cells in premature neonates (3) and recognize gram-negative lipopolysaccharide (LPS). Once bound to LPS, TLR4 activates pathogen-associated molecular pattern (PAMP) receptors further inducing the destruction of the gut barrier and allows for the observed intestinal permeability. The increased ability of bacterial translocation results in both a heightened innate and adaptive immune response, recruiting macrophages, dendritic cells, neutrophils, tumor necrosis factor-alpha (TNFα), IL-6 and other inflammatory cytokines. Additionally, the disruption of the balance between T helper 17 (Th17) cells and FoxP3+ regulatory T (Treg) cells further characterizes this inflammatory phenotype (43). In NEC, a decrease in Treg cells disrupts the equilibrium and increases proinflammatory Th17 cell response (44). However, when AFSCs are administered, TLR4-mediated signaling is inhibited (44) and inflammatory markers such as TNFα and IL-6 are significantly decreased (45).

### Stem cell and regeneration ability

The rescue of intestinal stem cell (ISC) function and differentiation is paramount to intestinal epithelial rescue and impairment as a lack of cell proliferation and differentiation grants further destruction of epithelial integrity. Leucine-rich repeat-containing G-protein coupled receptor 5 (Lgr5) is a well-studied stem cell proliferation marker. During homeostasis, Lgr5+ ISCs located at the crypt base are responsible for mediating the constant renewal of the intestinal epithelium. These stem cells undergo self-renewal to maintain an undifferentiated ISC reservoir, and differentiation to give rise to major intestinal epithelial cell types, such as absorptive enterocytes, goblet cells, Paneth cells, tuft cells and enteroendocrine cells (46).

In premature infants there is a decrease in Lgr5+ ISCs which inhibits intestinal epithelial reconstitution and increases NEC susceptibility (47). AFSC medicated intestinal recovery *via* Wnt signaling can be attributed to canonical Wnt pathways as demonstrated by blocking Wnt signaling *via* inhibitors of Porcupine (Porcn) protein (44). It has been well established that transforming growth factor β1 (TGFβ1) activates the canonical Wnt signaling (48). TGF-β1 is downregulated in NEC and has been suggested to be used as a biomarker for early diagnosis and to assess disease severity (49). Intestinal stem cell Lgr5+ is upregulated in the embryonic intestinal specimens (50) and gastrointestinal cancer (51) exposed to an exogenous TGFβ1, which is in line with studies demonstrating that AFSC upregulated the expression of Lgr5 through the Wnt signalling pathway.

The administration of AFSCs rescued ISC function, the differentiation of goblet cells is readily increased, thus the creation of mucins to restore gut function and protection (44). Thus, AFSCs ultimately promote intestinal recovery by activating the Wnt/β-catenin signaling pathway to increase Lgr5+ ISCs, reduce inflammation and regenerate the intestinal epithelium (44).

## AFSC derivatives in NEC

Although AFSCs improved safety, accessibility and ethical requirement of stem cell therapy, concerns continue to arise in clinical translation due to undesired differentiation, possible pro-tumorigenic and other detrimental effects (28). Thus, research has shifted its focus more to derivatives of the AFSC. For example, administration of the conditioned medium (CM) or extracellular vesicles (EVs) derived from AFSCs provide the same benefit as administering the stem cells themselves (52, 53).

### Conditioned Medium (CM)

CM is essentially a cell-free “soup” of factors secreted by cells, including growth factors, cytokines, enzymes, nucleic acids and bioactive lipids (54). However, a gap in knowledge remained whether CM cultured with AFSCs would provide the same alleviation in experimental NEC.

A recent study demonstrated that administration of human AFSC conditioned medium (hAFSC-CM) improved NEC survival and reduced intestinal injury (45). Specifically, intestinal mucosal inflammation and epithelial apoptosis were significantly reduced. Remarkably, hAFSC-CM restored intestinal regeneration capabilities *via* increased expression of epithelial proliferation and intestinal stem cell markers. In addition, intestinal microvasculature and angiogenesis was restored.

Moreover, the secretome of the hAFSC-CM was assessed to gain a further understanding of its composition to explain the beneficial effects within *in vivo* models of NEC. Over 1500 proteins were found in the CM, mostly enriched by exosomes. In addition, several clusters included immunomodulation, cell cycle and stem cell regulation, which is in line with study results previously described. Interestingly, a large functional cluster of this secretome analysis was vesicle-mediated transport, which led to the interest in the function of these extracellular vesicles in the context of NEC.

### Extracellular vesicles (EV)

The paracrine communication of AFSCs, as widely discussed in previous research findings, has now been accredited to the function of EVs (55–58). EVs are membrane-limited vesicles released from the outward budding of plasma membranes into the extracellular space with the capacity of intercellular communication and component exchange (59, 60). Some of these components include proteins and nucleic acids, reflecting the phenotype of parental cells (61). AFSC-CM, as previously described, has beneficial effects on experimental models of NEC, which include the paracrine function of these EVs. Recent studies went further and established that EVs alone were efficacious in the treatment of experimental NEC (9, 62).

Experimental NEC-induced intestinal damage was rescued *via* administration of human AFSC-derived EVs (hAFSC-EVs) (63). This study focused, however, on the most known affected intestinal area in experimental NEC, the ileum. In this experimental model, EVs improved ileal morphology, decreased markers ileal inflammation (IL-6; TNFα), increased ileal proliferation and regeneration by rescuing the stem cell niche, which is in line with their previous work on hAFSC-CM.

**Figure 3:**
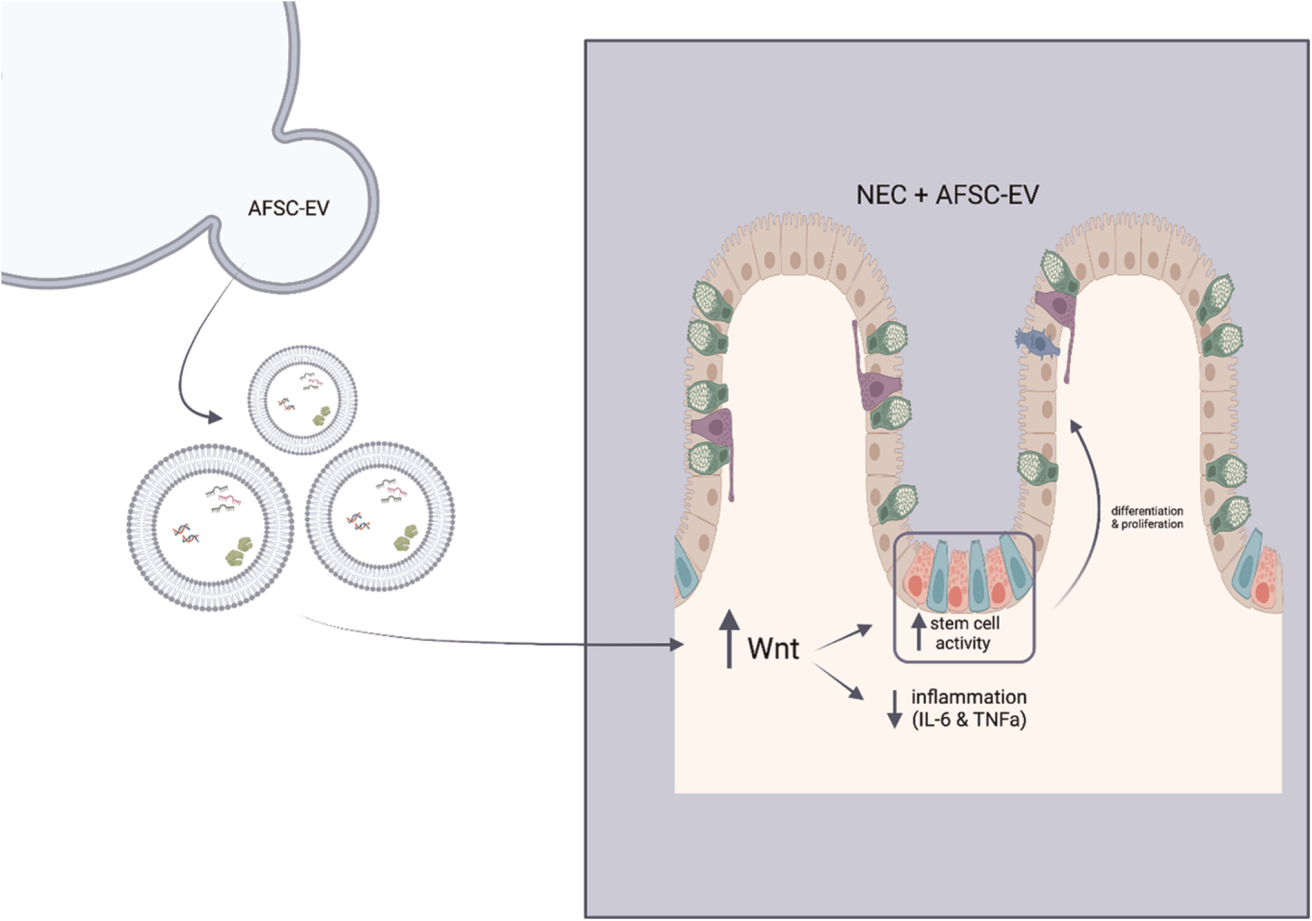
Schematic of mechanism of action of hAFSC-EVs. Wnt signalling becomes enhanced upon administration of hAFSC-EVs, which in turn increases stem cell activity and decreases inflammatory responses.

A recent study indicates that AFSC-EVs counteract NEC-induced intestinal injury through the stimulation of the Wnt/β-catenin signaling pathway (Figure 3) (44). It is known that intestinal stem cell (ISC) impairment is attributed to a lack of Wnt signaling in NEC pathogenesis (48). In an ex vivo NEC model, following hAFSC-EV administration, ISC and epithelial proliferation were increased *via* Wnt signaling in intestinal organoids (44). In an *in vivo* NEC model, Wnt-producing AFSCs administered to mouse pups were able to rescue epithelial injury, reduce inflammation and increase ISC and epithelial proliferation (44). However, Wnt-deficient AFSCs administered to mouse pups did not show these same results. Interestingly, AFSC-secreted factors were both able to increase ISC activity and Wnt signaling in a healthy and injured gut. Furthermore, when administering AFSC-EVs to both ex vivo and *in vivo* NEC models, similar results were observed as previously described. In addition, the authors concluded that timing is significant in EV-induced intestinal recovery, even though they were unable to prevent this injury (44).

## Challenges of clinical translation of AFSC-based therapy in NEC: What's next?

Overall, AFSC-based therapy has proven to be a novel therapeutic option in the context of experimental NEC. The pluripotent nature of these cells allows for their vast differentiation capabilities. In addition to its direct benefits to patients, the significant reduction of ethical issues of this less invasive tool in comparison to embryonic stem cells and other stem cell origins increases its attractiveness in tissue engineering and treatment of suffering neonates. Though there is a significant amount of evidence proving AFSCs to be valuable in the treatment of NEC, there remains a lack of clinical trials as indicated by our recent search in ClinicalTrials.gov.

Optimization of stem cell and stem cell derivative expansion is vital to clinical application. Moreover, various factors must be considered prior to the induction of experimental animal model techniques of AFSC administration, such as identifying: (1) the ideal gestational age to harvest AFSCs, (2) markers eliciting optimal cells, (3) an optimized standard for AFSC expansion (4) optimized dose concentrations of AFSC derivatives, (5) stem cell conditioning for enhanced applications, (6) proving that animal study results can be translated to a human setting without detrimental effects. In the following paragraphs, these factors are explained in more detail.

The general quality of AFSC cultures is significantly correlated with gestational age of the fetus when AF is harvested. In studies with experimental spina bifida patients, MSCs and neural SCs show to have been affected by gestational age (27). Recent studies with AFSCs show a similar trend, however gestational age is shown to affect paracrine signalling capabilities. For example, differential modulation of lymphocyte proliferation is most affected in the second trimester of pregnancy. Expression levels of HLA molecules and sensitivity to natural killer (NK) cell-mediated lysis are decreased, while T and NK cell proliferation is not efficiently inhibited but B cells are, which is not seen in first or third trimester cells (64). Immunomodulation, especially the suppression of inflammatory responses, is a meaningful attribute of AFSCs. Thus, the communication between lymphocytes and these cells must be optimized, and gestational age may allow for this to occur. While gestational age yields different effects, cell cultures stratified based on maternal age yielded no significant differences in the quality or repair capabilities (27).

On the quality of these cell cultures, AFSC morphology yield differential properties in protecting the neonate. There are two types of AFSCs: spindle-shaped and round-shaped. Those that are spindle-shaped possess greater neuroprotection to the developing fetal brain *via* analysis of CD90 and CD105. It is well studied that the consequences of NEC go on to affect neurodevelopment and brain morphology, therefore, these results support AFSC-mediated protection *via* the use of optimal cells (65). In addition, Oct-4 is a common biomarker of pluripotency capabilities and should be used when analyzing harvested hAFSC cultures. Optimizing the quality of cell cultures harvested *via* ideal gestational age and predictable cell markers will increase the confidence researchers have in the induction of AFSC cultures in clinical application. However, it remains unknown whether quality of these cell cultures is hindered when harvested from diseased sources. For example, harvesting AFSCs from a fetus with NEC. Baughn et al. studied whether the use of AFSCs harvested from patients with neural tube defects have differential quality or repair capabilities (27). Further research is needed to elucidate these implications, thus contributing to the lack of clinical AFSC induction.

As previously discussed, the use of CM and EVs are evidenced to be additional therapeutic tools. These EVs containing soluble bioactive factors and complex cargo have secretomes that differentially affect immune responses, including lymphocyte proliferation, and regeneration (64). EVs from different individuals contain various proteins that may not be present in their neighbour, thus elucidating minor differential effects. Therefore, results seen in many studies must consider how variance may play a significant factor in how clinical application is affected.

The culture medium in which cell expansion occurs has been optimized for *in vitro* use with animal models involving animal sera. The standardization of optimized 10%–15% fetal bovine serum (FBS) was studied in the clonal expansion of AFSC cultures, however, results yielded noncompliance with good manufacturing practice (GMP) (28, 66). Though AFSCs do not yield the same safety issues other stem cell lines do, it remains important to keep GMP-compliance when handling these cells to avoid all possibility of infectious disease transmission. For other stem cell lineages, xeno-free reagents have been used as animal sera substitute for clinical translation as xeno reagents induce immune rejection (66). However, platelet-derived products are now becoming the increasingly popular substitute. Platelets are rich in growth factors that modulate growth, repair and angiogenic capabilities. In addition, this type of media has the capacity to preserve cells involved in immunomodulation and the suppression of infectious disease transmission (28). Lyset, a commercial platelet-derived product, has been proven to be a safe alternative culture medium as it does not compromise the integrity and functionality of AFSCs in clinical translation (28).

In recent research, short-term small molecule treatments are a growing interest within the field of regenerative medicine. These molecules can improve stem cell characteristics to further reinforce their therapeutic potential and efficiency. A study assessed the effect of various concentrations and mixtures of small molecules known to participate in cell repair and regeneration on AFSCs, such as surface marker and pluripotency associated gene expression (67). HDAC inhibitors trichostatin A (TSA) and sodium butyrate (NaBut) promote somatic cell reprogramming, while multifunctional molecules retinoic acid (RA) and vitamin C (vitC) synergistically boost pluripotency. These small molecules in combination with each other have no cytotoxic effects but do increase gene expression patters of pluripotency markers and neurogenic transcription factors (Oct4, Nanog, Lin28a, Cmyc, Notch1, Sox2). Surface level markers are also affected (SSEA4, CD117, Tra-1-81, CD105) (67). Lastly, the effect on metabolic phenotype was significantly increased. Thus, it is plausible for the application of these small molecules as a pre-treatment strategy to maintain, even boost, AFSC functional efficiency.

To overcome these hurdles within this research field of AFSC-based therapy, key knowledge must further be elucidated prior to the implementation of randomized control trials in human neonates. Experimental NEC trials are currently completed in animal models, thus further investigation is required to investigate the effects of this therapy on human models, such as intestinal epithelial maturation, barrier function and innate immune responses. For example, working with human-derived organoids would be a great first step to preclinical human models (68).

## Conclusion

To conclude, we have identified important bodies of knowledge that continue to lack within the scope of clinical translation of AFSC-based therapy. Future research must confirm whether this treatment strategy will benefit neonates suffering from NEC *via* preclinical trials. This review provides a beneficial frame of reference towards clinical application of AFSC and/or its derivatives as a novel treatment against NEC.
